# Exploring potential mechanistic aspects of “Biaoben acupoint” acupuncture for lumbar disc herniation using 4D proteomics

**DOI:** 10.3389/fneur.2025.1630736

**Published:** 2025-09-02

**Authors:** Fudong Shi, Zhiyi Wu, Zuoxu Li, Jiao Jin, Hai Lin, Ning Liu, Guojun Wang, Chun Chen, Yadi Feng, Yuzhang Liu, Shimin Zhang

**Affiliations:** ^1^Wangjing Hospital, China Academy of Chinese Medical Sciences, Beijing, China; ^2^School of Traditional Chinese Medicine, Beijing University of Chinese Medicine, Beijing, China

**Keywords:** lumbar disc herniation, Biaoben acupoint, acupuncture, 4D-DIA quantitative proteomics, differentially expressed proteins

## Abstract

**Objective:**

To investigate the mechanism of action of “Biaoben acupoint” acupuncture in treating lumbar disc herniation (LDH) by identifying differentially expressed plasma proteins (DEPs) using timsTOF Pro-based 4D data-independent acquisition (DIA) proteomics and correlating them with clinical indicators.

**Methods:**

This study enrolled 10 healthy individuals (H group) and 10 patients diagnosed with LDH. Plasma samples were collected from LDH patients both before treatment (LDH group) and after three weeks of “Biaoben acupoint” acupuncture treatment (Acu group). Clinical outcomes, including Visual Analogue Scale (VAS) for pain, Oswestry Disability Index (ODI) for lumbar function, and Japanese Orthopaedic Association (JOA) score for neurological status, were assessed before and after treatment. Plasma samples were collected for proteomic analysis and key core proteins were further validated by ELISA.

**Results:**

Acupuncture treatment significantly improved VAS, ODI, and JOA scores in the Acu group compared to the LDH group (*p* < 0.001). Proteomic analysis quantified 3,685 proteins, identifying 376 DEPs across the healthy group and the experimental group (before and after treatment). Bioinformatics analysis revealed that these DEPs were primarily enriched in pathways related to cell structure and adhesion (e.g., cytoskeleton remodeling, focal adhesion), inflammation and immune signaling (e.g., chemokine and cytokine signaling), and cell signal transduction (e.g., calcium signaling, Rap1 pathway). Core DEPs included ACTB, CXCR4, ACTN1, CXCL12, SELP, and CCN2. Correlation analysis demonstrated that the expression levels of CXCL12, ACTN1, CXCR4, and CCN2 were significantly correlated with VAS, ODI, and JOA scores. To further validate these findings, ELISA was performed on plasma samples from all three groups. The results confirmed that CXCL12, CXCR4, and CCN2 levels were significantly elevated, while ACTN1 was decreased in the LDH group compared to healthy controls; these changes were reversed following acupuncture treatment, showing trends consistent with the proteomic data.

**Conclusion:**

“Biaoben acupoint” acupuncture likely exerts its therapeutic effects by modulating multiple biological pathways related to inflammatory/immune responses, cytoskeleton organization, cell structure/adhesion, and tissue repair, thereby improving pain, lumbar function, and neurological deficits in LDH patients. Proteins such as CXCL12, ACTN1, CXCR4, and CCN2 are potential key mediators of these therapeutic effects.

## 1 Introduction

Lumbar disc herniation (LDH) is a common degenerative spinal disorder primarily characterized by lower back pain, radiating pain in the lower extremities, and restricted mobility, leading to significant impairment in patients’ quality of life (1). In China, the incidence of LDH has been increasing annually—from approximately 7.62% in 2010 to approximately 15% in recent years. The resulting burden contributes to 15–30% of labor force loss, healthcare expenditure, and disability (2, 3). Currently, western medical treatments primarily include nonsteroidal anti-inflammatory drugs (NSAIDs), physical therapy, and surgical interventions. However, long-term medication may cause gastrointestinal side effects, while surgical procedures are associated with high costs, significant trauma, and postoperative complications (4). In contrast, acupuncture, a core modality of traditional Chinese medicine (TCM), offers advantages such as ease of administration, low incidence of adverse effects, and demonstrated clinical efficacy. Multiple clinical studies have confirmed its efficacy in alleviating LDH-associated pain and functional impairments (5).

Acupoint matching based on the “Biaoben” (symptom-root correspondence) theory is one of the important guiding principles for acupoint selection in acupuncture and moxibustion (6). Our research team has achieved significant clinical outcomes by applying this theory in LDH treatment. Specifically, Shenshu (BL23) and Dachangshu (BL25) are used as “symptom acupoints” to promote local circulation of Qi and blood, enhance regional microcirculation, and relieve lumbar muscle spasms. In parallel, Houxi (SI3), Weizhong (BL40), Huantiao (GB30), and Waiguan (SJ5) are selected as “root acupoints” to regulate the meridians and internal organ Qi dynamics, unblock pain pathways, modulate neural signaling, and improve lower limb function—thereby achieving comprehensive symptom-root coordination (7). However, existing studies have predominantly focused on clinical efficacy, while the molecular mechanisms—particularly the protein-level regulatory networks—remain poorly understood, limiting the scientific advancement and standardization of this acupuncture protocol.

In recent years, 4D proteomics has emerged as a promising approach to elucidate disease mechanisms due to its high resolution and sensitivity. The data-independent acquisition (DIA) strategy enables comprehensive capture of low-abundance proteins and allows precise characterization of proteomic alterations under pathological conditions. In this study, we applied 4D proteomic techniques in combination with clinical efficacy assessments to systematically investigate the molecular mechanisms underlying “symptom-root acupoint” acupuncture treatment of LDH. Our goal is to elucidate the multi-target, multi-pathway effects of acupuncture from the perspective of dynamic protein regulation and explore its potential therapeutic mechanisms for LDH, thereby providing a scientific basis for the modernization of traditional acupuncture.

## 2 Materials and methods

### 2.1 Study subjects

A total of 10 healthy volunteers were enrolled as the control group (H group), and 10 patients diagnosed with LDH who visited the Department of Spine, Wangjing Hospital, China Academy of Chinese Medical Sciences between August 2024 and November 2024 were enrolled as the experimental group. Each LDH patient provided two samples: one before acupuncture treatment (LDH group) and one after completing a 3-week “Biaoben acupoint” acupuncture protocol (Acu group). In total, 30 samples were collected for 4D DIA proteomic analysis. The healthy volunteers underwent lumbar spine MRI examinations confirming the absence of disc herniation and were age-matched with the patients in the LDH group.

**Figure 1:**
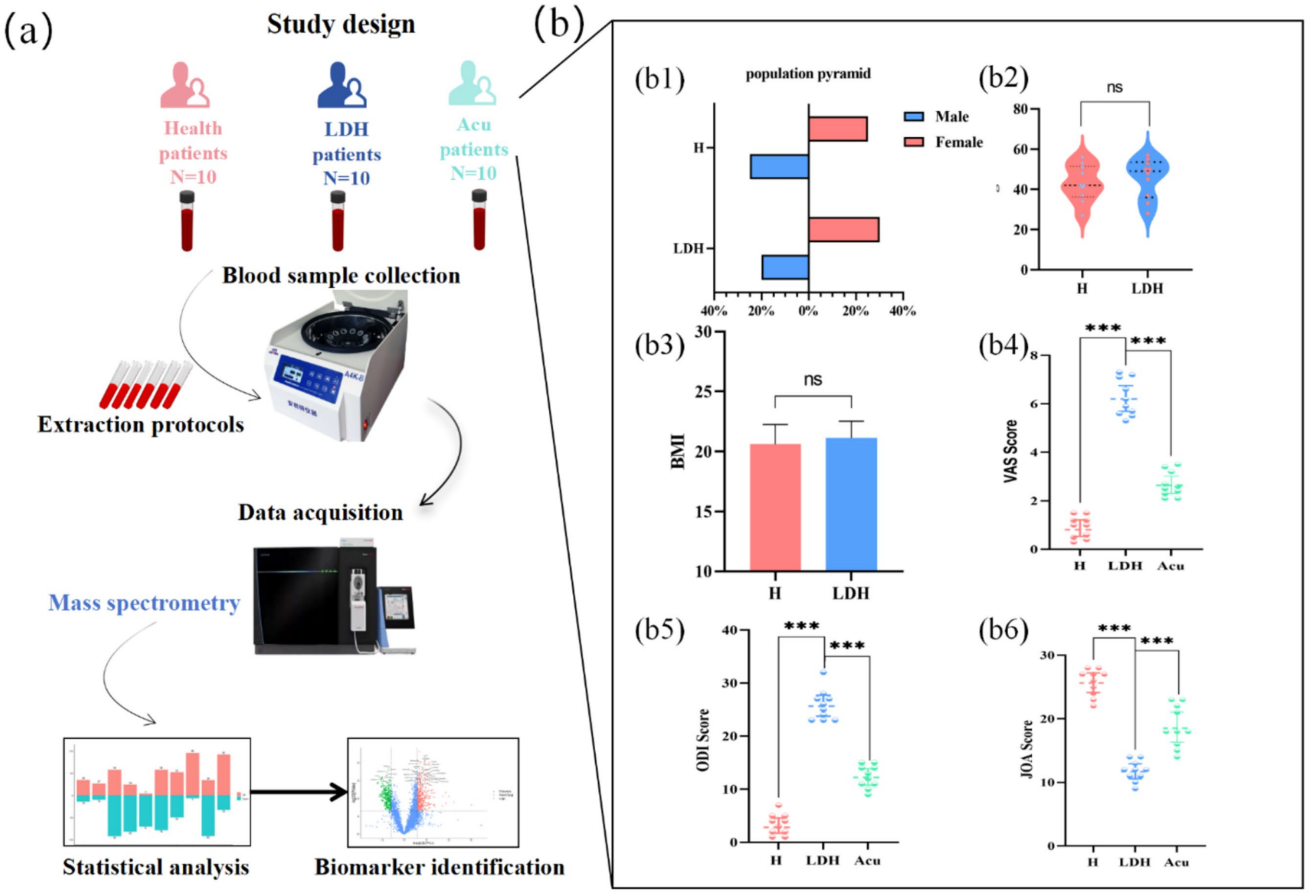
Study design and participant baseline characteristics. The study design process is shown in **(a)**. Baseline characteristics of all enrolled participants are presented in **(b)**. **(b1)** Sex distribution. **(b2)** Age distribution. **(b3)** BMI. **(b4)** VAS scores. **(b5)** ODI scores. **(b6)** JOA scores. *N* = 30. Comparisons with the LDH group are indicated as ^***^p < 0.001; ns, not significant; H, healthy control group; LDH, patients before acupuncture treatment; Acu, patients after three weeks of acupuncture treatment.

This study was approved by the Ethics Committee of Wangjing Hospital, China Academy of Chinese Medical Sciences (Approval No. WJEC-KT-2024-045-P002). Written informed consent was obtained from all participants. The diagnosis of LDH was confirmed by experienced clinicians based on clinical symptoms and lumbar MRI or CT imaging. The basic clinical parameters of the H group, the LDH group, and the Acu group are shown in Figure 1.

### 2.2 Diagnostic criteria and inclusion/exclusion criteria

Based on the clinical guidelines issued by the North American Spine Society (8), patients were diagnosed with LDH if the herniation site confirmed by lumbar MRI or CT was consistent with clinical symptoms. In addition, patients were required to meet at least 3 of the following diagnostic criteria (a–f): (a) A history of low back pain. (b) Radiating pain in the lower extremity along the distribution of the nerve root. (c) Sensory abnormalities in the lower limbs corresponding to the dermatome of the affected nerve. (d) Positive straight leg raising test accompanied by aggravation of symptoms. (e) Decreased tendon reflexes on the affected side compared to the healthy side. (f) MRI or CT showing disc herniation with nerve compression. Patients meeting any of the following inclusion criteria were eligible: (1) Male or female patients aged 18–60 years. (2) Presence of typical symptoms such as low back and leg pain, clearly diagnosed LDH with a VAS score ≥4. (3) Ability to understand and voluntarily sign informed consent for participation in the study. Patients who met any of the following exclusion criteria were withdrawn from the study within 24 h: (1) Significant osteophyte formation with severe lumbar spinal stenosis, lumbar spondylolisthesis, or isthmic spondylolysis. (2) Complications such as cauda equina syndrome, conus medullaris syndrome, or other absolute surgical indications. (3) Severe osteoporosis. (4) Severe skin damage, infection, or dermatological conditions at the treatment site. (5) Severe cardiovascular disease, endocrine disorders, or psychiatric conditions. (6) Pregnancy, lactation, or plans to conceive during the study period. (7) History of spinal surgery.

### 2.3 Intervention methods

The acupuncture treatment for patients in the experimental group was based on the national standard for acupoint location (9), including Dachangshu (BL25), Shenshu (BL23), and the following acupoints: Weizhong (BL40) and Huantiao (GB30) on the affected side, Houxi (SI3) and Waiguan (SJ5) on the healthy side. The treatment followed the principle of “treating the symptom before the root”: acupuncture was first applied to Dachangshu and Shenshu, followed by Huantiao and Weizhong on the affected side, and finally Houxi and Waiguan on the healthy side.

Disposable sterile acupuncture needles (0.25 mm × 50 mm) manufactured by HanYi, Tianjin, were used for all acupoints except Huantiao, for which longer needles (0.25 mm × 75 mm) were used. All needles were inserted perpendicularly at a 90° angle with balanced reinforcing and reducing techniques. The arrival of Qi was confirmed by the patient’s sensation of soreness, numbness, distention, heaviness, or radiating pain. After achieving the Qi response, the needles were retained for 30 min. Upon removal, sterile dry cotton swabs were used to apply gentle pressure to the needle sites to prevent bleeding.

Acupuncture was administered three times per week on alternate days to ensure both continuity of treatment and adequate recovery time. All procedures were performed by the same experienced acupuncturist to maintain consistency in technique and comparability of therapeutic effects. The treatment lasted for 3 weeks in total. The clinical status of the experimental group before and after treatment is presented in Figure 1.

### 2.4 Instruments, reagents, and consumables

The instruments used in this study included a RIGOL L-3000 high-performance liquid chromatography (HPLC) system (RIGOL Technologies Co., Ltd., Beijing, China), a vortex mixer (SCILOGEX, MX-S), a vacuum centrifugal concentrator (Beijing Giamother Technology Co., Ltd., CV100-DNA), an electric thermostatic water bath (Beijing Guangming Medical Instrument Co., Ltd., XMTD-7000), a centrifuge (Eppendorf), a microplate reader (DR200B), an electrophoresis system (Bio-Rad), a high-throughput tissue grinder (Shanghai Hefan Instrument Co., Ltd., HF-48), and an ultrasonic cell disruptor (Shanghai Huxi Industrial Co., Ltd., JY96-IIN). Key reagents and consumables included 10K ultrafiltration tubes (Sartorius, VN01H02), ammonium bicarbonate (Sigma-Aldrich, A6141-500G), TEAB buffer (Sigma-Aldrich, T7408-100 mL), urea (Amresco, M123-1KG), protein quantification dye (Huaxingbio, HXJ5137), bovine serum albumin (Thermo Scientific, 23209), dithiothreitol (DTT; Amresco, M109-5G), iodoacetamide (IAM; Amresco, M216-30G), trypsin (Promega, V5280/100 μg), Ziptip pipette tips (Millipore, ZTC18M096), acetonitrile (J.T. Baker, 34851 MSDS), ammonia solution (Wako Pure Chemical Industries Ltd., 013-23355), and formic acid (Sigma-Aldrich, T79708), as well as sample vials and caps (Thermo, 11190533 and 11150635, respectively). Proteomic sample preparation and enrichment were performed using QLBIO kits, including the MagicOmics-DMB8X plasma low-abundance protein enrichment kit, MagicOmics-EMB8X blood exosome enrichment kit, MagicOmics-MMB8X universal/micro protein preparation kit, and the MagicOmics-AP-96 automated proteomics workstation.

ELISA kits used in the study included: Human SDF-1 (Stromal Cell Derived Factor 1) ELISA Kit, Catalog No. EH3755, Wuhan Fine Biotech Co., Ltd. (Wuhan, China); Human CXCR4 (C-X-C chemokine receptor type 4) ELISA Kit, Catalog No. EH2136, Wuhan Fine Biotech Co., Ltd. (Wuhan, China); Human CTGF (Connective Tissue Growth Factor) ELISA Kit, Catalog No. EH0702, Wuhan Fine Biotech Co., Ltd. (Wuhan, China); Human ACTN1 (Alpha-actinin-1) ELISA Kit, Catalog No. EH4480, Wuhan Fine Biotech Co., Ltd. (Wuhan, China).

### 2.5 Sample collection

Fasting venous blood samples (4 mL) were collected from the antecubital vein of all participants in a seated position in the early morning using disposable EDTA anticoagulation tubes. After collection, the blood was gently mixed by inverting the tubes 8 to 10 times and then left to stand at 4 °C. The samples were centrifuged at 600 × g for 10 min to separate plasma. The supernatant (plasma) was carefully transferred into EP tubes and stored at −80 °C until subsequent proteomic analysis and ELISA assays for validation of key core proteins.

### 2.6 Protein extraction, digestion, desalting, and library construction

A volume of 300 μL of 8 M urea was added to each sample, along with a protease inhibitor cocktail at 10% of the lysis buffer volume. The mixture was centrifuged at 14,100 × g for 20 min, and the supernatant was collected. Protein concentration was determined using the Bradford assay, and the remaining samples were stored at −80 °C for future use.

For digestion, 100 μg of extracted protein was reduced with 200 mM dithiothreitol (DTT) at 37 °C for 1 h. The sample was then diluted fourfold with 25 mM ammonium bicarbonate (ABC) buffer, followed by the addition of trypsin at a 1:50 enzyme-to-protein ratio and incubated overnight at 37 °C. Digestion was terminated the next day by adding 50 μL of 0.1% formic acid (FA).

C18 columns were prewashed repeatedly with 100 μL of 100% acetonitrile (ACN) and 0.1% FA before sample loading. Peptides were eluted using 70% ACN, and the eluates were pooled and freeze-dried, then stored at −80 °C.

Peptide fractionation was performed on a Waters BEH C18 column (4.6 × 250 mm, 5 μm) using a Rigol L3000 high-performance liquid chromatography (HPLC) system at a flow rate of 1 mL/min and a column temperature of 50 °C. The eluent was monitored at 214 nm, with one fraction collected per minute and ultimately combined into six fractions. These fractions were vacuum-dried and reconstituted in 0.1% (v/v) FA aqueous solution. For retention time calibration in downstream analysis, 0.2 μL of iRT standard peptides (iRTkit, Biognosys) was spiked into the peptide solution.

Proteomic analysis was performed in data-dependent acquisition (DDA) mode using an EASY-nLC^™^ 1200 UHPLC system coupled with an Orbitrap Q Exactive HF-X mass spectrometer (Thermo Fisher Scientific) (10).

### 2.7 LC-MS/MS analysis

After peptide fractionation, samples were analyzed in data-dependent acquisition (DDA) mode using a quadrupole-Orbitrap mass spectrometer (Q Exactive HF-X, Thermo Fisher Scientific) coupled with an EASY-nLC 1200 system. A total of 500 ng of peptides per sample was loaded onto a 25 cm analytical column (inner diameter: 150 μm), and separated using a gradient elution as follows: 8–12% buffer B over 7 min, 12–30% buffer B over 48 min, and finally 95% buffer B over 15 min. The total run time was 80 min, with the column maintained at 60 °C. MS spectra were acquired in a “top-40” DDA mode, with MS1 and MS2 resolutions set at 120,000 and 15,000, respectively.

For data-independent acquisition (DIA) analysis of tissue samples, a total of 42 variable windows were used to cover a mass range of 350–1,500 m/z. The AGC (automatic gain control) target was set to 3 × 10^6^, the maximum injection time was 80 ms, and the dynamic exclusion time was 16 s.

### 2.8 Protein identification and quantification

Fractionated pooled samples (DDA MS data from six fractions) and individual subject samples (DIA MS data) were used to construct a hybrid spectral library in Spectronaut software (Biognosys, version 15.7.220308.50606). This library was subsequently used to search the DIA data for comprehensive protein identification and quantification.

All searches were conducted against the human UniProt reference proteome database (including canonical and isoform sequences; total 20,373 entries, downloaded in March 2022). Carbamidomethylation was set as a fixed modification, while protein N-terminal acetylation and methionine oxidation were specified as variable modifications. Other search parameters followed default settings. Trypsin/P was used as the digestion enzyme, allowing a maximum of two missed cleavages, and the peptide length was limited to 7–52 amino acids.

Protein intensity normalization was performed using Spectronaut’s built-in local normalization algorithm based on a locally weighted regression model. For spectral library construction, each peptide was required to have at least three fragment ions, with a maximum of six top-ranked fragments selected. The false discovery rate (FDR) for both protein and precursor levels was controlled at 1%. Quantitative results were reported only for proteins that passed all quality filtering criteria (11).

### 2.9 Raw data processing and differential analysis

Raw data were normalized using the median method to eliminate experimental error. Data with more than 50% missing values in any sample were excluded, and missing values were imputed using the KNN algorithm. Differential analysis was performed using the Mann–Whitney *U* test. Differentially expressed proteins (DEPs) were identified based on the following criteria: a fold change (FC) of ≥1.5 or ≤0.67 and a *p*-value <0.05. Benjamini–Yekutieli correction was applied to control the false discovery rate (FDR) at <5%.

### 2.10 Bioinformatics analysis and visualization

Functional enrichment analysis of DEPs was performed based on their structural and functional annotations from various databases, including Gene Ontology (GO) and the Kyoto Encyclopedia of Genes and Genomes (KEGG). Over-representation analysis (ORA) was used to assess enrichment, with statistical significance evaluated via the hypergeometric distribution. Enrichment *p*-values were calculated, followed by false discovery rate (FDR) correction for multiple hypothesis testing. Enrichment scores were calculated as −log(*p*-value), where smaller *p*-values or FDR values indicate stronger enrichment, implying greater biological relevance of the corresponding pathway in the dataset.

The formula for the hypergeometric test is as follows:


$$\begin{array}{l} p-\text{value}=\sum_{i=m}^{M}\frac{\left(\begin{array}{l} M\\ i \end{array})(\begin{array}{l} N-M\\ n-i \end{array}\right)}{\left(\begin{array}{l} N\\ n \end{array}\right)}\;\\ \;=1-\sum_{i=0}^{m-1}\frac{\left(\begin{array}{l} M\\ i \end{array})(\begin{array}{l} N-M\\ n-i \end{array}\right)}{\left(\begin{array}{l} N\\ n \end{array}\right)} \end{array}$$

*N*: total number of genes in the annotation database; *M*: number of genes annotated to a specific functional category; *n*: total number of DEPs in the study; *m*: number of DEPs annotated to that specific category.

Protein family classification and pathway analysis were conducted using the COG (Clusters of Orthologous Groups), KEGG,^1^ and Reactome^2^ databases (12). GO annotation was derived from the GO database^3^ and categorized into molecular function (MF), biological process (BP), and cellular component (CC). KEGG pathway enrichment was statistically assessed using the hypergeometric distribution (13). Protein–protein interaction (PPI) networks were constructed using the STRING database (http://string.embl.de/, confidence score >0.7). Topological analysis of the PPI network was performed using the CytoNCA plugin in Cytoscape, and the top 20 hub genes were identified based on betweenness centrality for further visualization.

### 2.11 Correlation analysis between core proteins and key clinical indicators

To explore the relationship between candidate protein expression and clinical phenotypes in LDH patients, Spearman’s rank correlation analysis was performed between the top 10 core candidate proteins identified from the post-treatment PPI network and clinical assessments including the Visual Analogue Scale (VAS) for pain, Oswestry Disability Index (ODI) for lumbar function, and the Japanese Orthopedic Association (JOA) score for lumbar neurological function. Spearman’s correlation is suitable for non-normally distributed data and effectively captures potential nonlinear relationships. Correlation coefficients (Rho) and significance *p*-values were calculated using a custom get_cor() function, and results were tabulated. To visually represent the strength of correlations, a heatmap was generated using the pheatmap package, where color intensity reflects the absolute correlation coefficients. Furthermore, scatter plots combined with boxplots were created via the get_scatter_cor() function to illustrate protein expression distributions across different clinical phenotypes. To ensure methodological rigor, robustness, and reproducibility, experimental data were structured and managed using the Summarized Experiment object framework.

### 2.12 Enzyme-linked immunosorbent assay validation test

The concentrations of CXCL12, CXCR4, CCN2, and ACTN1 in the samples were quantified using enzyme-linked immunosorbent assay (ELISA) kits purchased from Wuhan Fine Biotech Co., Ltd. (Wuhan, China), following the manufacturer’s protocol. Briefly, 100 μL of either standard solution or sample was added to each well of the microplate, sealed with adhesive film, and incubated at 37 °C for 90 min. The plate was then washed twice without soaking. Subsequently, 100 μL of biotin-conjugated antibody working solution was added to each well, followed by incubation at 37 °C for 60 min. The plate was then washed three times, with each wash involving a 1-min soak. Next, 100 μL of HRP-conjugated streptavidin (SABC) working solution was added and incubated at 37 °C for 30 min, followed by five washes with 1-min soaks each time. Thereafter, 90 μL of TMB substrate solution was added to each well, and the plate was incubated at 37 °C for 10–20 min in the dark. The reaction was terminated by adding 50 μL of stop solution, and the optical density (OD) was immediately measured at 450 nm. Protein concentrations were calculated based on the standard curve.

Due to the small sample size and non-normal distribution of the ELISA data, non-parametric tests were used: the Mann–Whitney *U* test for comparisons between the H and LDH groups, and the Wilcoxon signed-rank test for paired comparisons between the LDH and Acu groups. *p* < 0.05 was considered statistically significant.

## 3 Results

### 3.1 Study design and participants

Blood samples were collected from the patient cohort at Wangjing Hospital of the China Academy of Chinese Medical Sciences, including a total of 20 subjects and 30 plasma samples for 4D DIA quantitative proteomics analysis. The study was conducted strictly in accordance with the experimental protocol approved by the Ethics Committee of Wangjing Hospital of the China Academy of Chinese Medical Sciences (Figure 1). According to the inclusion and exclusion criteria, basic demographic and clinical data were collected from patients enrolled in each study group. These data included sex, age, body mass index (BMI), as well as VAS (Visual Analogue Scale), ODI (Oswestry Disability Index), and JOA (Japanese Orthopedic Association) scores (Figure 1), which were used for clinical diagnosis of LDH and evaluation of acupuncture treatment efficacy (Figures 1b1–b6). There were no statistically significant differences in sex, age, or BMI among the groups (*p* > 0.05). Significant differences were observed in VAS, ODI, and JOA scores between the LDH group and the H group (*p* < 0.001), indicating marked pain and functional impairment in LDH patients; significant differences were also found between the Acu group and the LDH group (*p* < 0.001), suggesting that acupuncture treatment effectively reversed pain and functional impairment in LDH patients. The collection of all baseline information in this study fully complied with the requirements of the Ethics Committee.

### 3.2 Plasma proteomics analysis

#### 3.2.1 Quality control of DIA data and visualization of global identification results

Data retrieval was performed using the built-in Pulsar search engine in Spectronaut, establishing a spectral library based on both DDA and DIA data. The library identified 84,344 peptides and 7,028 proteins. DIA protein identification yielded 44,116 peptides and 3,685 proteins.

**Figure 2:**
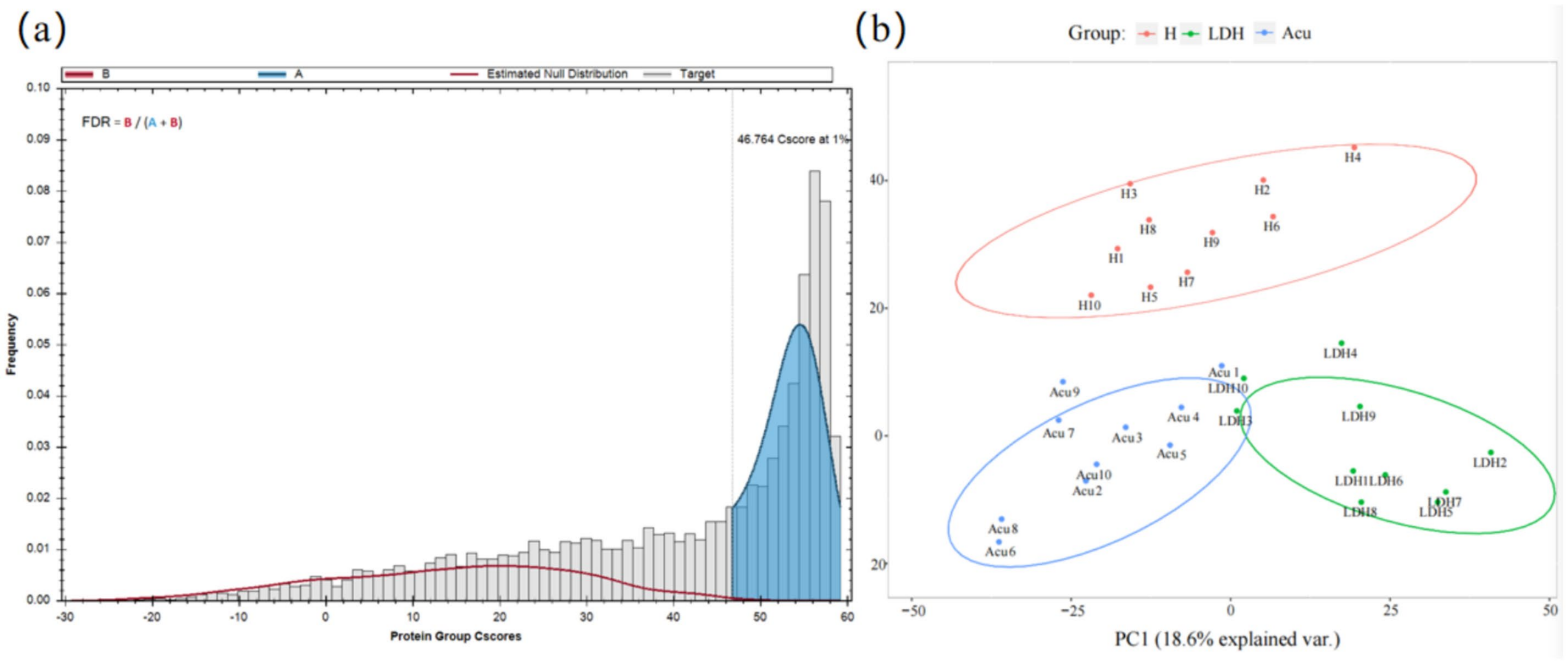
Data quality control and visualization of global identification results. **(a)** Protein FDR plot: the gray histogram represents the distribution of target proteins, i.e., truly identified proteins. The red curve (estimated null distribution, B) represents the estimated null hypothesis distribution, i.e., false positive distribution. B (red area) corresponds to incorrectly identified proteins (false positives), while A (blue area) corresponds to correctly identified proteins (high-confidence identifications). FDR = B/(A + B) indicates the proportion of false identifications, with a threshold set at 1%. **(b)** PCoA plot: samples of the same color belong to the same group. The closer the samples cluster together, the smaller the intra-group variability and the better the reproducibility. Conversely, greater distances between samples indicate larger differences among them.

A false discovery rate (FDR) threshold of 1% was applied to filter scoring thresholds and evaluate the reliability of identification results (Figure 2a). The results showed that when the Cscore ≥46.764, the FDR was controlled below 1%, indicating reliable identifications. The blue area was much larger than the red area, demonstrating high-quality experimental data. The distribution of target proteins (gray) was clearly distinguishable from the false positive distribution (red), indicating accurate protein identification.

Following mass spectrometry analysis of blood samples and removal of missing values, principal coordinate analysis (PCoA) was performed on the proteomic data to reflect the clustering of DEPs (Figure 2b). PCoA is a method based on ranking a series of features or vectors to visualize data similarity and investigate biological differences or variability among LDH patient groups at the proteomic level. This approach enables intuitive assessment of inter-group differences and intra-group reproducibility, as well as identification of outliers. Results showed that the LDH, H, and Acu groups were well separated with considerable differences among groups, while intra-group distributions were relatively clustered, indicating good reproducibility.

#### 3.2.2 Analysis of DEPs

**Figure 3:**
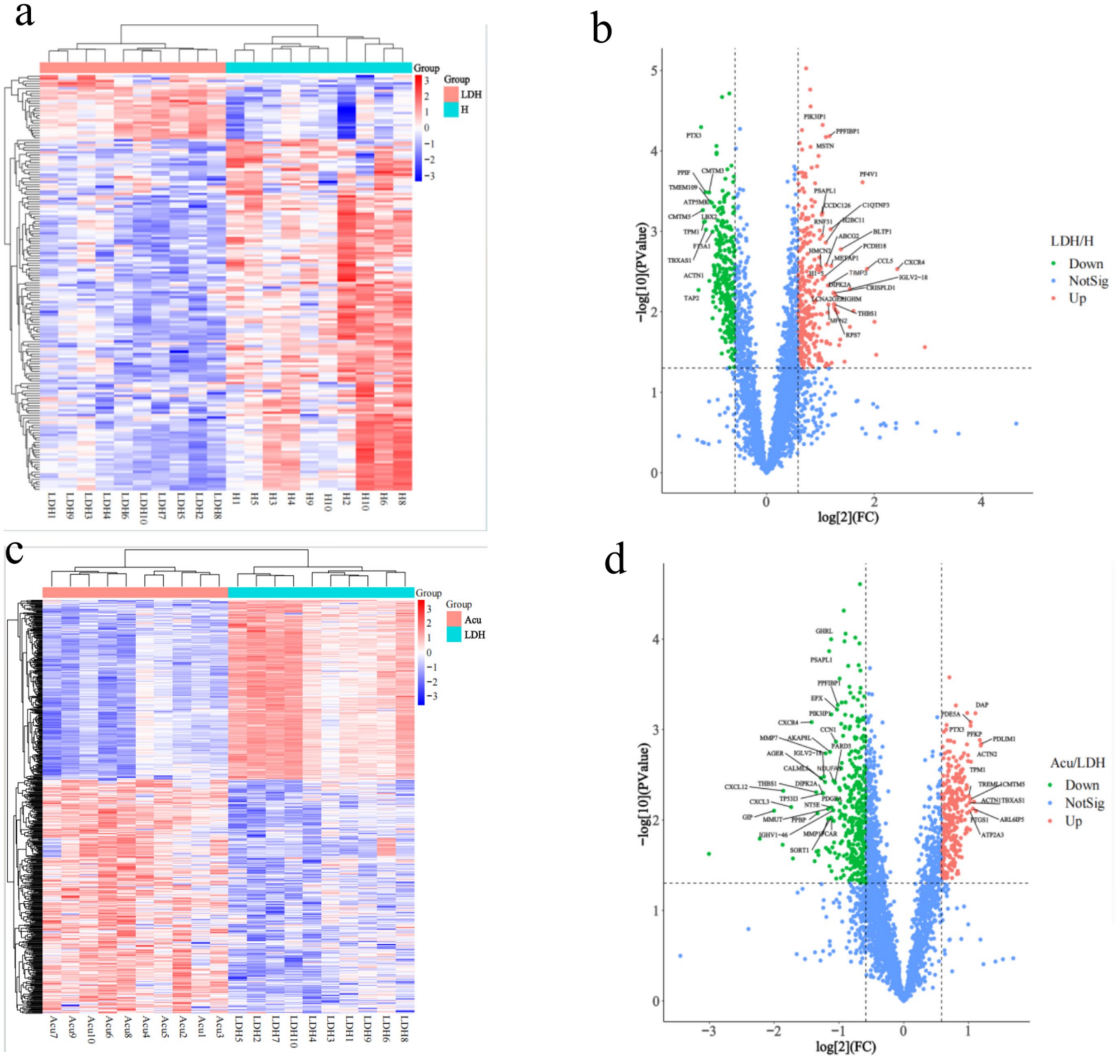
Clustering analysis of DEPs. H, healthy control group; LDH, patients before acupuncture treatment; Acu, patients after 3 weeks of acupuncture treatment. **(a,c)** Represent hierarchical clustering heatmaps of DEPs between the LDH and H groups, and between the Acu and LDH groups, respectively (horizontal axis: individual samples; vertical axis: DEPs). **(b,d)** Show volcano plots of DEPs comparing the LDH and H groups, and the Acu and LDH groups, respectively. In the volcano plots, the two vertical dashed lines indicate fold change (FC) thresholds, corresponding to FC <1/1.5 and FC >1.5. The horizontal dashed line indicates the *p*-value threshold (*p* < 0.05). Red dots in the upper right corner represent significantly upregulated DEPs, green dots in the upper left represent significantly downregulated DEPs, and blue dots represent proteins with no significant change.

To investigate the molecular protein characteristics before and after treatment in LDH patients, DEPs were screened under the criteria of ratio >2 or ratio <0.5 and adjusted *p*-value <0.05. Results revealed significant differences in protein expression between the LDH group and the H group (Figure 3a). The volcano plot identified a total of 603 DEPs (312 upregulated and 291 downregulated) (Figure 3b), further illustrating the protein expression differences between the LDH and H groups.

Analysis of DEPs between the Acu group and LDH group identified 659 DEPs (286 upregulated and 373 downregulated). The clustering heatmap of DEPs showed that the protein expression pattern of the post-treatment group gradually approached that of the H group (Figure 3c), suggesting a possible reversal of abnormal protein expression following treatment. The volcano plot further confirmed the regulatory effect of treatment on protein expression (Figure 3d).

In summary, significant differences in protein expression were observed between the LDH group and the H group, while the expression profile of the Acu group showed a trend toward normalization, approaching that of healthy controls.

#### 3.2.3 Bioinformatic analysis and visualization

##### 3.2.3.1 GO enrichment analysis

To explore the potential biological functions and underlying mechanisms of DEPs in LDH, Gene Ontology (GO) enrichment analysis was performed across three categories: molecular function (MF), biological process (BP), and cellular component (CC). The enrichment results of GO-CC and GO-MF were visualized using a chord clustering diagram. In this diagram, the innermost ring displays a clustering dendrogram based on the log fold change (LogFC) values of DEPs, the middle ring shows the expression levels of individual DEPs (red indicating upregulation and blue indicating downregulation), and the outermost ring annotates the enriched GO terms. Additionally, a horizontal bar chart was used to illustrate the GO-BP enrichment results, with separate analyses for upregulated and downregulated proteins. The x-axis represents “−log10(*p*-value),” where higher values indicate stronger associations between the biological process and the differentially expressed proteins.

**Figure 4:**
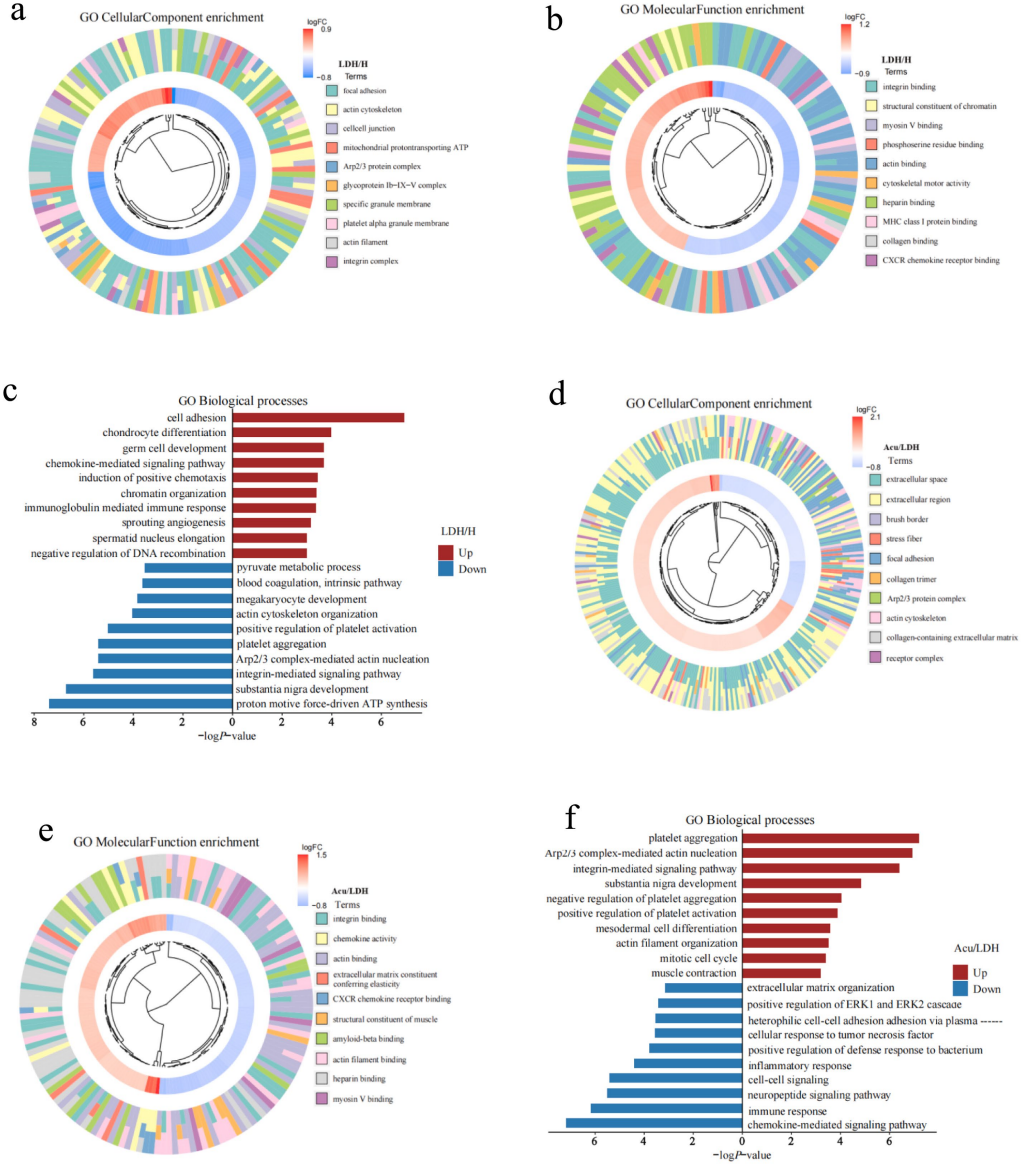
GO functional enrichment analysis of DEPs. **(a–c)** Present the GO-CC (cellular component), GO-MF (molecular function), and GO-BP (biological process) enrichment results of DEPs between the pre-treatment LDH group and the healthy control group. **(d–f)** Show the corresponding GO-CC, GO-MF, and GO-BP enrichment results for DEPs between the Acu group and the LDH groups.

Compared with the H group, GO enrichment analysis of DEPs in the LDH group revealed (Figures 4a–c) disrupted cytoskeletal integrity, dysregulated energy metabolism, reduced cell adhesion, and aberrant activation of inflammatory responses and chemokine signaling pathways, suggesting the presence of multifaceted cellular dysfunctions in the LDH pathological state.

Further analysis of functional changes in DEPs between the LDH and Acu groups (Figures 4d–f) showed that acupuncture markedly upregulated proteins associated with cytoskeletal remodeling, muscle contraction, tissue repair, and integrin signaling, while downregulating proteins related to inflammatory responses, chemokine-mediated signaling, and abnormal intercellular communication. These findings suggest that acupuncture may exert therapeutic effects by restoring cellular structural stability, regulating extracellular matrix interactions and signaling pathways, and suppressing inflammatory cascades.

##### 3.2.3.2 KEGG enrichment analysis

To explore the potential signaling pathways involved in the pathogenesis of LDH and the therapeutic effects of acupuncture, KEGG pathway enrichment analysis was performed on the DEPs. The top 10 significantly enriched pathways were visualized using bubble plots, which displayed the number and significance of DEPs associated with each pathway: each circle represents an enriched pathway, with larger circles indicating a greater number of associated proteins, and deeper red colors indicating higher enrichment significance [represented as −log₁₀(*p*-value)]. Additionally, horizontal bar plots were used to present the top 10 enriched KEGG pathways for both upregulated and downregulated proteins.

Compared with the H group, DEPs in the LDH group were significantly enriched in pathways such as ECM-receptor interaction, cytokine–cytokine receptor interaction, PI3K-Akt signaling, and leukocyte transendothelial migration. These findings suggest that LDH may be closely associated with extracellular matrix remodeling, inflammatory activation, and immune cell infiltration. In contrast, downregulated proteins were mainly enriched in pathways such as tight junction and focal adhesion, potentially reflecting impaired cell barrier integrity under pathological conditions.

**Figure 5:**
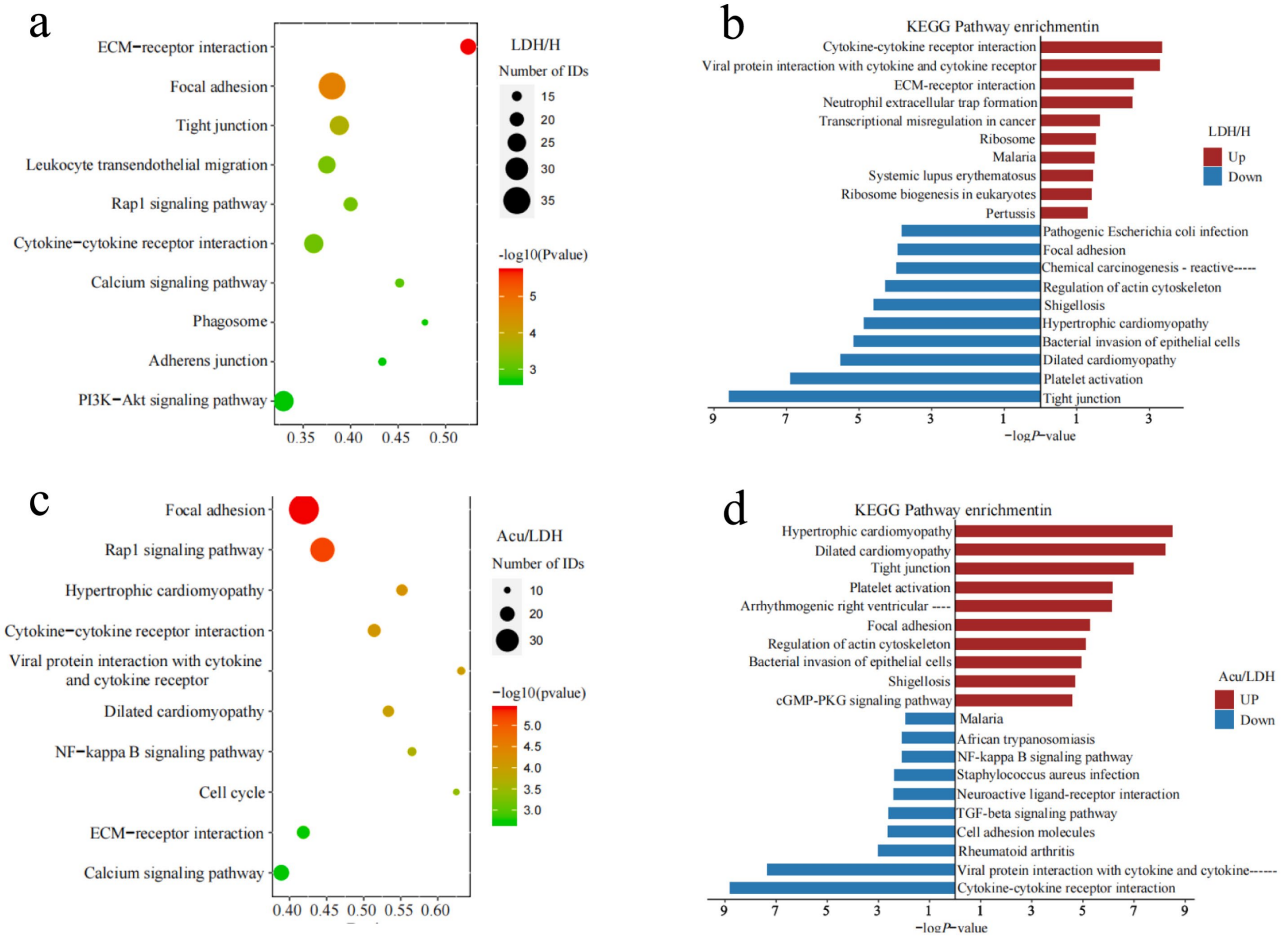
KEGG pathway annotation and functional enrichment analysis of DEPs. **(a,b)** Show the KEGG enrichment analysis results comparing the LDH group before treatment with the healthy control group. **(c,d)** Display the KEGG enrichment analysis results comparing the LDH group after acupuncture treatment with the same group before treatment.

In the comparison between the LDH and Acu groups, upregulated proteins in the Acu group were significantly enriched in pathways related to tight junctions, platelet activation, and focal adhesion, indicating that acupuncture may promote tissue repair by enhancing cell–cell connectivity and stabilizing barrier functions. Downregulated proteins were enriched in inflammation- and neuroactivation-related pathways, including cytokine signaling, calcium signaling, ECM-receptor interaction, Rap1 signaling, and the NF-κB pathway. These findings suggest that acupuncture may exert its analgesic and regulatory effects by suppressing inflammatory responses, alleviating neuroactivation, and improving the extracellular microenvironment (see Figure 5).

#### 3.2.4 Interaction network analysis of DEPs

**Figure 6:**
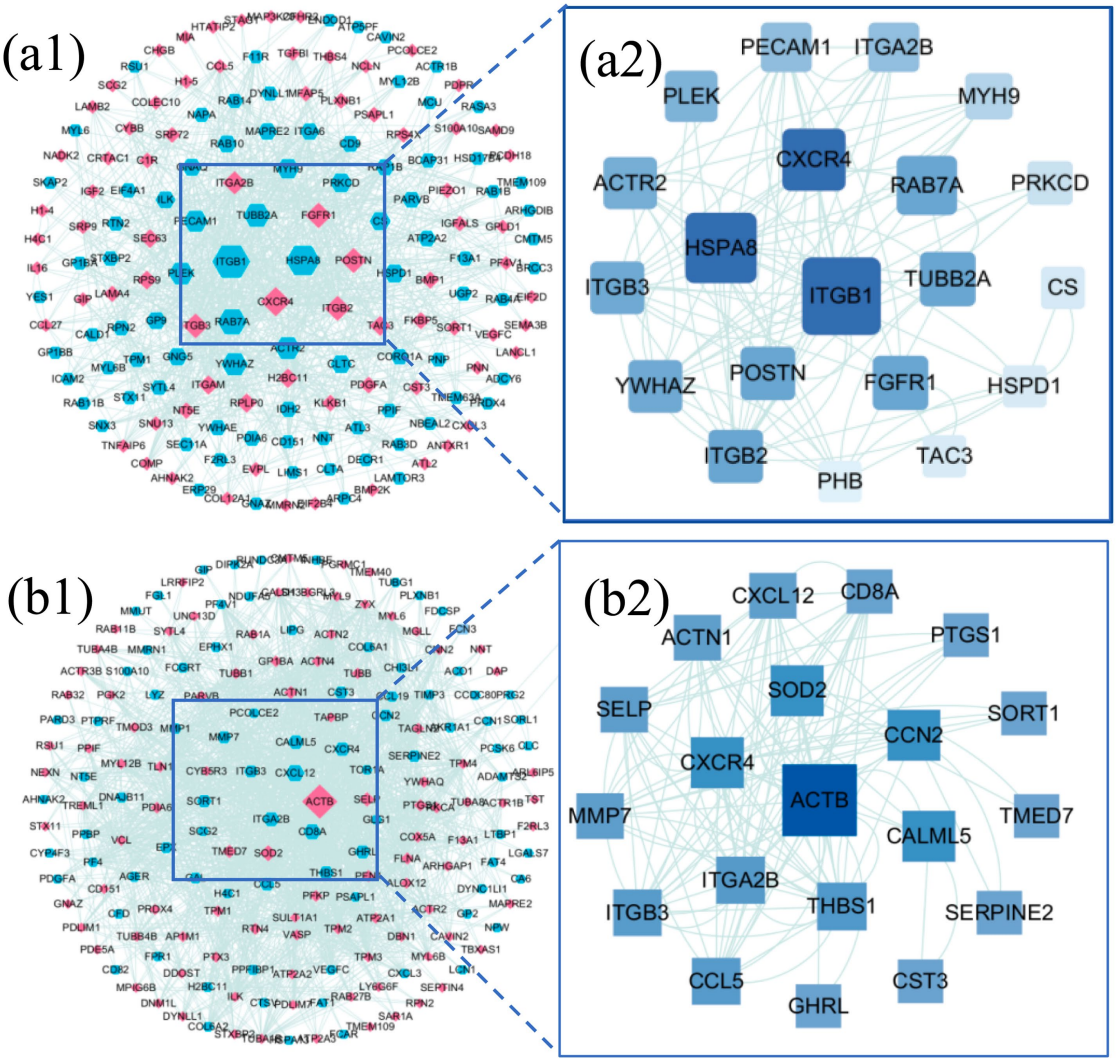
Interaction network analysis of DEPs. **(a1)** PPI network of DEPs between the LDH group (pre-treatment) and the healthy control group (H). **(a2)** Hub subnetwork extracted from the PPI network in **(a1)**, highlighting key hub proteins. **(b1)** PPI network of DEPs between the Acu group (post-acupuncture treatment) and the LDH group (pre-treatment). **(b2)** Hub subnetwork extracted from the PPI network in **(b1)**, showing the core hub protein interactions.

To further elucidate the core regulatory roles of DEPs within protein–protein interaction (PPI) networks, the top 100 significantly upregulated and downregulated DEPs from both the LDH vs. H group and the Acu vs. LDH group were selected to construct PPI networks. Topological characteristics of the networks were analyzed using the CytoNCA plugin in Cytoscape to compute betweenness centrality for each node, with visual representation shown in Figures 6a1,b1. In these visualized networks, node shapes differentiate types of DEPs—hexagons and diamonds represent upregulated and downregulated DEPs, respectively. Node color indicates expression trend, with red denoting upregulation and blue denoting downregulation. Edges represent known or predicted protein–protein interactions. The size of each node positively correlates with its betweenness centrality value; a higher value suggests a more prominent “mediating” role in the network, indicating that the protein may occupy a critical position in signaling pathways.

Based on betweenness centrality scores, the top 20 hub genes with high centrality were identified, and subnetworks were extracted for further analysis. In the comparison between the LDH and H groups (Figure 6a2), core hub genes included ITGB1, HSPA8, CXCR4, and RAB7A. In the comparison between the Acu and LDH groups (Figure 6b2), hub genes included ACTB, CXCR4, SOD2, and CCN2. These findings suggest that these proteins may play key regulatory roles in the pathogenesis of LDH and in the therapeutic mechanism of acupuncture based on the “syndrome-acupoint matching” theory. They are therefore of high biological significance and potential therapeutic relevance.

#### 3.2.5 Correlation analysis between core proteins and key clinical indicators

**Figure 7:**
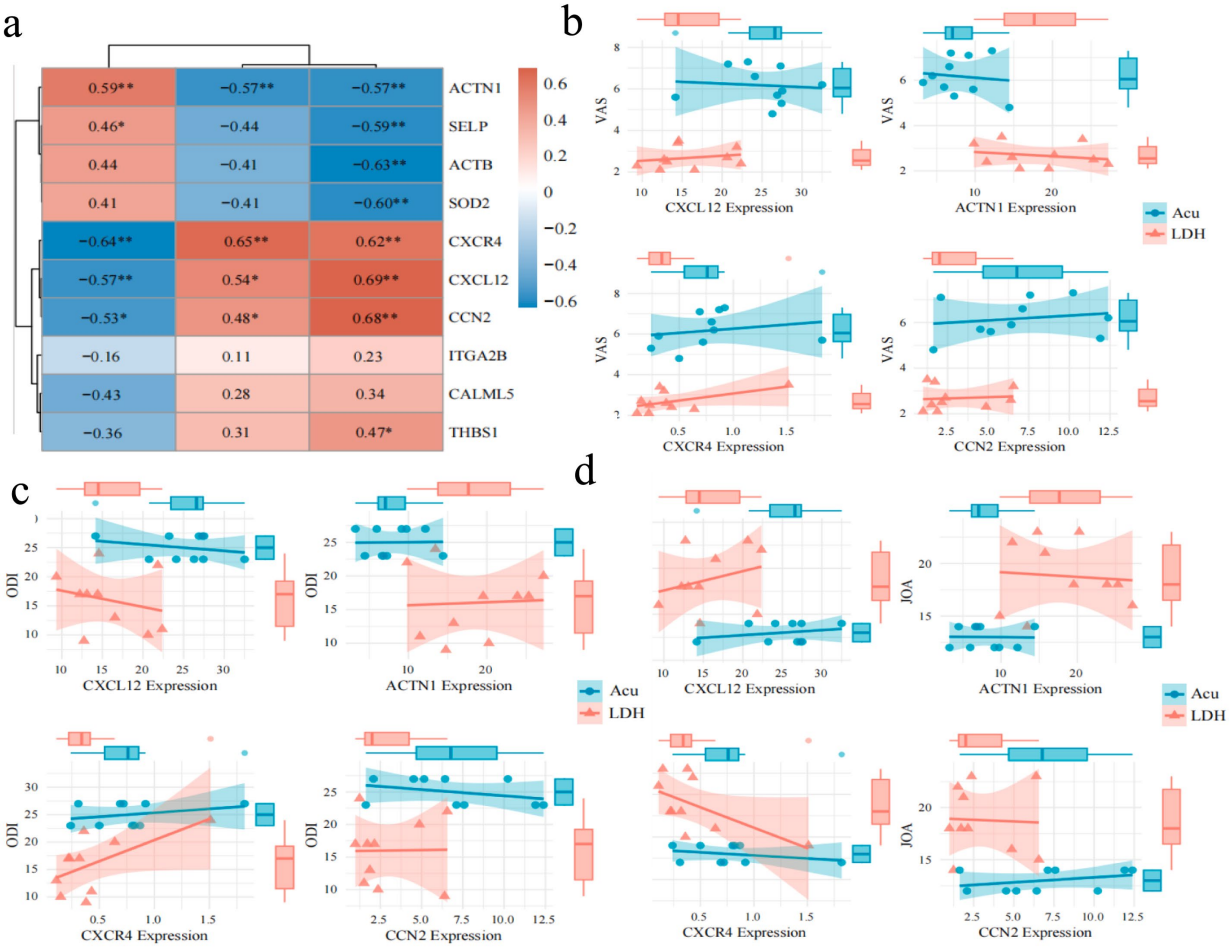
Spearman correlation analysis between key clinical indicators and protein expression patterns. **(a)** Heatmap of Spearman correlations between key core proteins and VAS, ODI, JOA scores before and after treatment in the experimental group (color intensity indicates correlation strength; red denotes positive correlation, blue denotes negative correlation; “*” indicates *p* < 0.05, “**” indicates *p* < 0.01). **(b)** Correlation analysis and group comparisons of core proteins CXCL12, ACTN1, CXCR4, and CCN2 with VAS scores. **(c)** Correlation analysis and group comparisons of core proteins CXCL12, ACTN1, CXCR4, and CCN2 with ODI scores. **(d)** Correlation analysis and group comparisons of core proteins CXCL12, ACTN1, CXCR4, and CCN2 with JOA scores.

To investigate the relationship between core proteins and key clinical indicators in LDH, a Spearman correlation analysis was conducted between the top 10 ranked proteins identified from the PPI network and the clinical indices VAS, ODI, and JOA (Figure 7a). The results indicated that CXCL12, ACTN1, CXCR4, and CCN2 were significantly correlated with all three clinical parameters. These four proteins with significant correlations were further visualized using correlation dependency plots (Figures 7b–d). In addition, several candidate proteins, including ACTB, SOD2, and SELP, showed significant negative correlations with VAS; however, their correlations with JOA and ODI were relatively weak and not statistically significant.

### 3.3 Validation of biomarkers

To further validate the feasibility of the biomarker candidates identified through 4D proteomics and correlation analysis, we performed ELISA assays on plasma samples from the three patient groups. Given the significant associations between CXCL12, CXCR4, CCN2, ACTN1 and clinical outcomes (VAS, ODI, JOA), these four proteins were selected for experimental validation.

The results showed that plasma levels of CXCL12, CXCR4, and CCN2 were significantly elevated in the LDH group compared to the H group (*p* < 0.001), while ACTN1 levels were significantly decreased. Notably, after acupuncture treatment, the Acu group exhibited a significant reduction in CXCL12, CXCR4, and CCN2 levels, alongside a marked increase in ACTN1 expression relative to the LDH group (*p* < 0.05).

**Figure 8:**
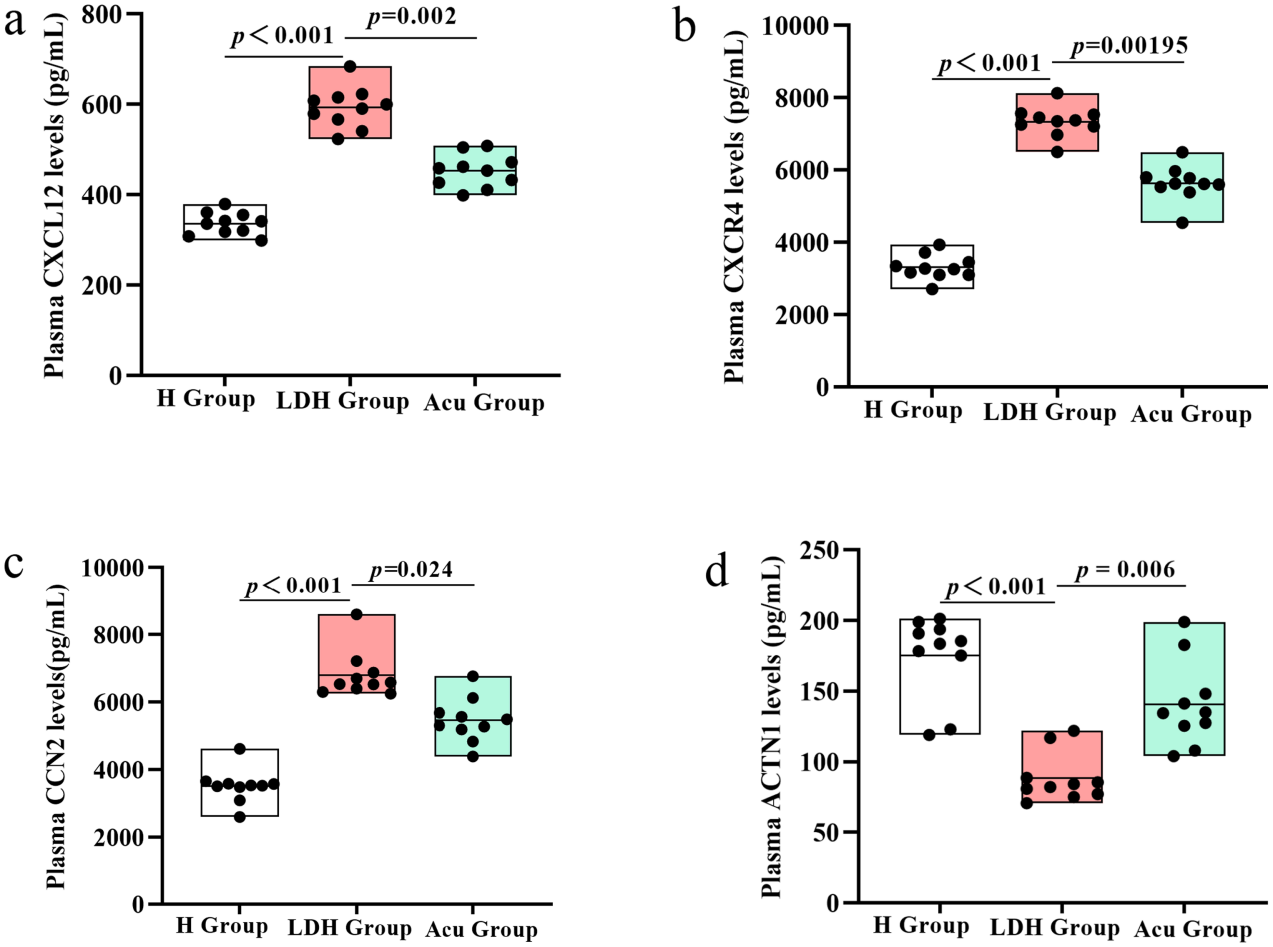
ELISA-based validation of core proteins CXCL12, CXCR4, CCN2, and ACTN1 in plasma samples from the H, LDH, and Acu groups. **(a)** Plasma CXCL12 levels and group comparisons among the H, LDH, and Acu groups. **(b)** Plasma CXCR4 levels and group comparisons among the H, LDH, and Acu groups. **(c)** Plasma CCN2 levels and group comparisons among the H, LDH, and Acu groups. **(d)** Plasma ACTN1 levels and group comparisons among the H, LDH, and Acu groups. Statistical significance was determined using the Mann–Whitney *U* test (H vs. LDH) and the Wilcoxon signed-rank test (LDH vs. Acu). *p* < 0.05 was considered statistically significant.

These findings provide additional experimental support for the proteomics results, and suggest that the four proteins—particularly CXCL12, CXCR4, and CCN2—may serve as key pro-pathogenic markers, while ACTN1 may act as a protective or restorative factor. Their modulation following acupuncture further implicates them in the therapeutic mechanism of action of “Biaoben acupoint” acupuncture in LDH (see Figure 8).

## 4 Discussion

In this study, 4D proteomics combined with bioinformatics analysis was employed to systematically investigate the potential molecular mechanisms underlying acupuncture treatment for LDH based on the “Biaoben acupoint” (symptom-root acupoint pairing) theory. Key proteins significantly associated with core clinical indicators—including the VAS, ODI, and JOA scores—were identified. Key proteins significantly associated with clinical outcomes—including VAS, ODI, and JOA scores—were identified. Patients in the Acu group exhibited significant improvements in low back and leg pain, functional disability, and neurological recovery after 3 weeks of acupuncture, accompanied by substantial modulation of protein expression profiles. These findings provide both clinical and molecular evidence supporting the efficacy of the “Biaoben acupoint” approach.

Proteomic analysis revealed widespread dysregulation in the plasma proteome of LDH patients compared to healthy controls. Following acupuncture treatment, 659 proteins showed significantly altered expression levels, with overall patterns in the Acu group trending toward normalization. To further validate the reliability of the proteomic data, ELISA assays were conducted on four key proteins—CXCL12, CXCR4, CCN2, and ACTN1—which were strongly correlated with clinical indicators. ELISA results confirmed that CXCL12, CXCR4, and CCN2 were significantly elevated in LDH patients and decreased after acupuncture, whereas ACTN1 was downregulated in LDH but significantly upregulated post-treatment. These findings reinforce the consistency between proteomic and biochemical data, highlighting these proteins as potential mechanistic biomarkers of acupuncture efficacy.

Pathway enrichment analysis further revealed that DEPs were significantly enriched in pathways related to focal adhesion, Rap1 signaling, cytokine–cytokine receptor interaction, ECM–receptor interaction, calcium signaling, and the NF-κB pathway. PPI network analysis identified CXCL12, CXCR4, CCN2, ACTN1, ACTB, and SELP as hub proteins potentially mediating acupuncture’s effects.

A salient finding of this investigation is the profound modulation of pathways governing inflammation and immune signaling. Sciatica induced by LDH is primarily attributed to the mechanical compression of nerve roots by the herniated nucleus pulposus. However, in clinical practice, the severity of nerve root compression does not always correlate with patients’ symptoms or pain scores, suggesting that mechanical compression alone may not fully account for the genesis of pain (14). An increasing body of evidence indicates that the local neuroinflammatory response triggered by herniated disc tissue plays a crucial role in the pathogenesis of LDH-associated pain (15, 16). The CXCL12/CXCR4 signaling axis has been extensively validated as a key regulator in both inflammatory responses and the initiation and maintenance of neuropathic pain (17, 18). The CXCL12/CXCR4 axis can activate several classical downstream inflammatory signaling pathways, including ERK1/2, PI3K-Akt, MAPK, and NF-κB. This activation subsequently promotes the release of proinflammatory factors, induces neuronal sensitization, and thereby facilitates both the generation and maintenance of pain (19). In this study, KEGG enrichment analysis following acupuncture intervention revealed widespread suppression of inflammation-related pathways, such as the cytokine signaling pathway, NF-κB pathway, and Rap1 pathway. Consistently, GO annotation indicated negative regulation of key biological processes, including chemokine-mediated signal transduction and ERK1/2 cascade activation. Moreover, previous studies have demonstrated that elevated levels of CXCL12 are positively correlated with the severity of chronic pain (20, 21). In agreement with these findings, the present study observed significantly elevated plasma levels of CXCL12 and CXCR4 in LDH patients, which were markedly reduced following acupuncture treatment. These expression trends were confirmed by both 4D proteomics and ELISA assays, providing robust evidence for the reliability of the observed molecular changes. Furthermore, the expression levels of these proteins were significantly correlated with VAS scores (CXCL12: *r* = 0.69, *p* < 0.01; CXCR4: *r* = 0.62, *p* < 0.01), supporting their functional relevance in pain modulation. These results suggest that inhibition of the CXCL12/CXCR4 signaling axis may constitute a key mechanism through which acupuncture—guided by the “syndrome-location acupoint matching” theory—alleviates neuropathic pain in LDH patients.

Additionally, the upregulation of SELP following acupuncture—accompanied by a negative correlation with VAS scores—suggests its dual role in immune cell trafficking and tissue repair (22). This implies that acupuncture may facilitate a transition from inflammation to tissue regeneration through enhanced cellular adhesion and vascular remodeling.

Beyond the suppression of inflammation, our data illuminate a concurrent promotion of biological processes related to tissue repair and structural integrity. In the PPI network analysis, ACTB emerged as the protein with the most extensive interaction profile, while ACTN1 was identified as a key hub protein with strong correlations to clinical parameters. Previous studies have shown that ACTB plays a critical role in neuronal structure formation, contributing to axon guidance, synaptic transport, and regeneration (23–25). ACTN1, a crucial regulator of cell adhesion and motility, governs pseudopodia formation and directional migration (26). In LDH patients, ACTN1 was markedly downregulated and subsequently restored following acupuncture, as confirmed by both proteomic analysis and ELISA validation. KEGG enrichment revealed that both ACTB and ACTN1 were involved in pathways such as tight junctions, platelet activation, focal adhesion, and actin cytoskeleton regulation after treatment. These findings suggest that acupuncture may promote neural and structural repair by enhancing cytoskeletal organization and cellular connectivity. However, whether acupuncture exerts its effects by directly improving the mechanical stability of disc cells, and the specific mechanisms involved, require further targeted studies—such as *in vitro* cellular biomechanics experiments—for clarification.

Previous studies have demonstrated that CCN2 plays a pivotal regulatory role in the progression of intervertebral disc degeneration. As a key downstream effector of the TGF-β signaling pathway, CCN2 mediates pathological processes such as angiogenesis, fibrosis, and discogenic low back pain (27). Painful intervertebral discs are often characterized by inflammation, neovascularization, and nucleus pulposus fibrosis, with higher CCN2 expression observed in painful discs compared to asymptomatic degenerated discs (28). In this study, CCN2 was significantly elevated in LDH patients and downregulated following acupuncture, as confirmed by both proteomic analysis and ELISA validation. KEGG enrichment analysis revealed a corresponding downregulation trend in the TGF-β pathway after treatment. Furthermore, CCN2 levels were positively correlated with VAS (*r* = 0.68, *p* < 0.01) and ODI (*r* = 0.48, *p* < 0.05), and negatively correlated with JOA scores (*r* = −0.53, *p* < 0.05), supporting its role as a potential biomarker mediating the analgesic and functional benefits of acupuncture in LDH.

In addition, thrombospondin-1 (THBS1), which was positively correlated with VAS scores (*r* = 0.47, *p* < 0.05), plays a role in extracellular matrix (ECM) construction and remodeling. It regulates angiogenesis inhibition, cell adhesion, and synaptic plasticity by activating the TGF-β pathway and modulating CD36/CD47-mediated signaling (29). The synergistic effects of THBS1 and CCN2 in maintaining ECM homeostasis and regulating cell adhesion and migration may contribute to the analgesic and reparative effects of acupuncture in LDH.

## 5 Limitations

This study has several limitations. First, although statistical power analysis was conducted to ensure adequate sensitivity, the relatively small sample size may still affect the generalizability of the findings. Second, while key proteins identified by 4D proteomics were validated using ELISA, further experimental studies—such as western blotting, immunohistochemistry, and *in vivo* functional assays—are required to confirm their biological roles and mechanistic relevance. Third, this study lacked an independent validation cohort and complementary multi-omics data (such as transcriptomics or metabolomics), which are essential for assessing the reproducibility of findings across different populations and for providing a more comprehensive, systems-level understanding of the molecular pathways involved.

## 6 Conclusion

This study systematically investigated the therapeutic mechanisms of “Biaoben acupoint” acupuncture for lumbar disc herniation (LDH) using 4D proteomics combined with ELISA validation. Acupuncture significantly alleviated pain and improved functional outcomes in patients. Proteomic analysis, supported by ELISA, identified key proteins—CXCL12, CXCR4, CCN2, and ACTN1—that were closely associated with clinical improvements. These proteins were enriched in pathways related to inflammation, cytoskeletal remodeling, and tissue repair, suggesting they may serve as critical molecular mediators of acupuncture’s effects. These findings provide molecular-level evidence supporting the clinical efficacy of “Biaoben acupoint” acupuncture and offer potential targets for future mechanistic and translational research.

## Data Availability

The original contributions presented in the study are publicly available. This data can be found here: https://www.iprox.cn/page/project.html?id=IPX0013082000.

## Footnotes

^1^ http://www.kegg.jp/kegg/pathway.html

^2^ https://reactome.org/

^3^ https://www.ebi.ac.uk/QuickGO/
